# TERT Extra-Telomeric Roles: Antioxidant Activity and Mitochondrial Protection

**DOI:** 10.3390/ijms24054450

**Published:** 2023-02-23

**Authors:** Jessica Marinaccio, Emanuela Micheli, Ion Udroiu, Michela Di Nottia, Rosalba Carrozzo, Nicolò Baranzini, Annalisa Grimaldi, Stefano Leone, Sandra Moreno, Maurizio Muzzi, Antonella Sgura

**Affiliations:** 1 Department of Science, University “ROMA TRE”, 00146 Rome, Italy; 2 Unit of Muscular and Neurodegenerative Disorders, Laboratory of Molecular Medicine, Bambino Gesù Children’s Hospital IRCCS, 00146 Rome, Italy; 3 Department of Biotechnology and Life Sciences, University of Insubria, 21100 Varese, Italy; 4 Department of Anatomical, Histological, Forensic Medicine and Orthopedics Sciences, Sapienza University of Rome, 00161 Rome, Italy; 5 IRCCS Santa Lucia Foundation, 00179 Rome, Italy

**Keywords:** telomerase catalytic subunit, mitochondrion, oxidative response, primary cell lines, electron microscopy

## Abstract

Telomerase reverse transcriptase (TERT) is the catalytic subunit of telomerase holoenzyme, which adds telomeric DNA repeats on chromosome ends to counteract telomere shortening. In addition, there is evidence of TERT non-canonical functions, among which is an antioxidant role. In order to better investigate this role, we tested the response to X-rays and H_2_O_2_ treatment in hTERT-overexpressing human fibroblasts (HF-TERT). We observed in HF-TERT a reduced induction of reactive oxygen species and an increased expression of the proteins involved in the antioxidant defense. Therefore, we also tested a possible role of TERT inside mitochondria. We confirmed TERT mitochondrial localization, which increases after oxidative stress (OS) induced by H_2_O_2_ treatment. We next evaluated some mitochondrial markers. The basal mitochondria quantity appeared reduced in HF-TERT compared to normal fibroblasts and an additional reduction was observed after OS; nevertheless, the mitochondrial membrane potential and morphology were better conserved in HF-TERT. Our results suggest a protective function of TERT against OS, also preserving mitochondrial functionality.

## 1. Introduction

Telomerase is a specialized reverse transcriptase, with the main function of adding telomeric DNA repeats on chromosome ends in order to counteract telomere shortening and maintain the stability of linear chromosomes [1]. Telomere shortening is caused by the so-called “end replication problem”, which is due to the inability of DNA polymerases to completely replicate the ends of a linear chromosome [2,3]. Furthermore, short telomeres can undergo different events such as degradation of the terminal regions of chromosomes or action of nuclease and other destructive factors [4]. In order to counteract telomere shortening, in humans, the main mechanism of telomere length maintenance involves the enzyme telomerase, a ribonucleoprotein complex consisting of two subunits: a functional catalytic protein called telomerase reverse transcriptase (TERT) and an RNA component (TERC), which works as a template site for DNA elongation [5,6]. The telomerase complex also contains a variety of accessory proteins essential for its biogenesis and function in vivo [7,8,9].

TERT and TERC are essential subunits for the correct functioning of telomerase [10,11]. However, while TERC is constitutively expressed in most somatic cells and in germ cells, TERT is considered a limiting factor because its expression is tightly regulated. Consequently, telomerase expression is finely controlled during embryogenesis and in most differentiated cells, where its activity is low or absent. Indeed, the protein is only expressed in somatic cells with rapid renewal potential, such as hematopoietic and stem cells [12,13].

Telomerase reactivation in normal human cells confers unlimited proliferative ability and may cause genomic instability, with a high probability of developing cancer [14,15]. Telomerase is active in 85% of tumor cells, while the other 15% displays a different mechanism of telomere length maintenance based on recombination, known as Alternative Lengthening of Telomere (ALT) [16,17].

In the last 20 years, several authors have reported that TERT is involved in mechanisms other than telomere maintenance [18], such as gene expression regulation of WNT/β-catenin or NF-kB pathways [19,20]. Its role in crucial physiological processes, including cell cycle, metabolism, differentiation, cell signaling and cell survival, has been recognized [19,21,22,23], even though interest in TERT functions has mainly been referred to anti-apoptotic [24,25] and antioxidant effects [26,27], besides protection against specific DNA-damaging agents [28,29,30]. 

The enhanced resistance to oxidative stress observed in TERT-overexpressing cells could be related to increased levels of the ROS scavenger glutathione (GSH), as well as of manganese superoxide dismutase (MnSOD or SOD2) and forkhead-box-protein O3 (FoxO3a) proteins [25,26,31].

The nuclear export of TERT is strictly controlled and such regulation includes shuttling between the nucleus and cytoplasm through nuclear export signal (NES), identified at the protein C-terminus. Upon oxidative stress, Scr kinase is the main trigger of the nuclear exclusion of TERT responsible for phosphorylation on tyrosine 707 [32]. In addition, a specific N-terminal sequence, comprising 20 amino acid residues represents the mitochondria transport signal (MTS) [33]. MTS is indispensable for TERT transport via the translocases of outer (TOM 20 and TOM 40) and inner mitochondrial membranes (TIM 23) and for its localization in the mitochondrial matrix, suggesting an active mitochondrial import mechanism [34,35]. Moreover, several authors have shown that hydrogen peroxide treatment increases TERT mitochondrial levels [33,34,36]. The significance of such induction remains unclear, as evidences of either exacerbation of H_2_O_2_-induced mtDNA damage or protection against different types of insult have been provided [33,37]. In fact, most data suggest that TERT participates in antioxidant response, to protect mtDNA, reduce mitochondrial ROS production and improve mitochondrial functions [36]. Moreover, the mechanisms underlying such action and the biological role of TERT in mitochondria remain undefined and require further investigation, focusing on this issue. 

In this research, we aimed to study TERT telomere-independent functions in a normal cellular context, taking advantage of normal human primary fibroblasts (HFFF2), cells without any mutation and that lack TERT expression and, consequently, telomerase activity. After the stable overexpression of the TERT protein in HFFF2 cells, we investigated the response to X-rays and H_2_O_2_ treatment, in terms of DNA damage and oxidative stress induction. Subsequently, we focused on the antioxidant response with a particular focus on TERT’s role inside mitochondria. Reactive oxidative species (ROS) level and the expression of factors commonly involved in the antioxidant response were measured. TERT translocation inside mitochondria was demonstrated in this work, and its consequences on the health and morphology of this compartment were investigated. 

## 2. Results

### 2.1. HF-TERT Cells Respond Better to Physical and Chemical Agent Treatments

In order to unravel the role of TERT in responding to different genotoxic agents, specifically X-rays and H_2_O_2_ treatment, we employed normal human primary fibroblasts (HFFF2), lacking TERT expression and consequent telomerase activity [13] and hTERT-overexpressing HFFF2 cells (HF-TERT). This cell line was obtained by retroviral transduction with a plasmid containing the cDNA for the human TERT and was tested by Western blot and RQ-TRAP (Real-time Quantitative—Telomeric Repeat Amplification Protocol assay) showing TERT expression and telomerase activity (Figure S1).

In the first instance, we compared the cell growth curves of normal and transduced fibroblasts subjected alternatively to X-rays and H_2_O_2_ treatment, in order to analyze the difference in cell proliferation due to TERT overexpression. As expected, even in untreated conditions HF-TERT displayed a higher growth rate than HFFF2. X-ray irradiation appeared to significantly affect cell proliferation in both cell lines, particularly in HFFF2 that, until 72 h, appeared to have no growth. On the contrary, H_2_O_2_ treatment led to a slight decrease in growth but not in a significant manner compared to control samples (Figure S2A,B). 

Comparing the different cell lines under the same treatment, it is possible to note that HFFF2 cells showed slower growth rates than HF-TERT after X-rays, differentially of hydrogen peroxide treatment in which there is no differences after treatment (Figure S2C,D).

**Figure 1:**
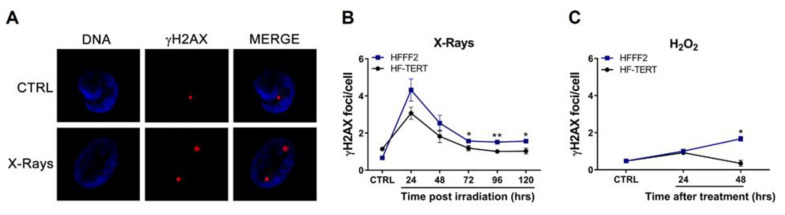
Detection of genomic DNA damage induced by X-ray irradiation or H_2_O_2_ treatment. (**A**) Representative images of immunofluorescence staining of γH2AX (red spot) in HF-TERT cells, before (CTRL) and after X-rays irradiation (X-rays). (**B**,**C**) Graphs evidencing data about the frequency of γH2AX after irradiation and hydrogen peroxide treatment. Bars correspond to standard error of γH2AX. Statistical analysis is performed between HFFF2 and HF-TERT cells. * *p* < 0.05; ** *p* < 0.01 by *t*-test.

To evaluate DNA damage induction after X-rays and H_2_O_2_ treatment, we detected by immunofluorescence a marker of DNA damage, the phosphorylated form of histone H2AX (γH2AX) that is recruited not only after a DNA double-strand break (DSB) but also due to replication fork stalling (Figure 1A) [38,39]. In HFFF2 and HF-TERT cell lines, both genotoxic agents induced a significant increase in γH2AX foci (Figure 1B,C). After X-rays irradiation in HFFF2 cells, a higher damage level persisted for many days after treatment while HF-TERT displayed less DNA damage, which was completely repaired in the following days (Figure 1B). After H_2_O_2_ treatment, both cell lines showed a similar behavior up to 24 h, while after 48 h HF-TERT completely repaired the genomic damage, differently from normal fibroblasts, in which we observed a further increase in γH2AX foci (Figure 1C). 

Since telomeric DNA is less efficiently repaired than other parts of the genome [40], and telomeric sequences are characterized by high content of guanine residues, these regions are more susceptible to oxidative damage, especially to the accumulation of oxidized bases such as 8-oxoguanine [41,42,43]; the presence of 8-oxoG is able to induce replication fork arrest at telomeres, resulting in DSB [43]. Thus, we analyzed a marker of telomeric dysfunction, TIFs (Telomere Induced Dysfunctional Foci) by immunofluorescence, detecting the co-localization of TRF1, a specific telomeric protein, with γH2AX (Figure S3A) [44]. In this case, we noted different levels of TIFs induction between the two cell lines. HF-TERT cells had also less telomeric damage both after X-rays and after H_2_O_2_ treatment compared to HFFF2 cells (Figure S3B,C).

Following TIFs results, we performed Quantitative-Fluorescence “In Situ” Hybridization (Q-FISH), a technique used to evaluate telomere length modulation (Figure S4A,B). X-rays caused modulation in telomere length in HFFF2 (as previously described in our lab, [45]) but not in HF-TERT (Figure S4C–E). This could be observed not only from the mean telomere lengths but above all from the shape of the telomere length distributions analyzed at 72 and 96 h after irradiation (Figure S4D,F). In addition, considering that telomere length stability in HF-TERT is probably due to telomerase activity, we performed RQ-TRAP assay to analyze telomerase activity after X-rays treatment. The data did not show any change in telomerase activity, which seems to be not affected by the treatment and probably not connected with the DNA damage level (Figure S4G).

**Figure 2:**
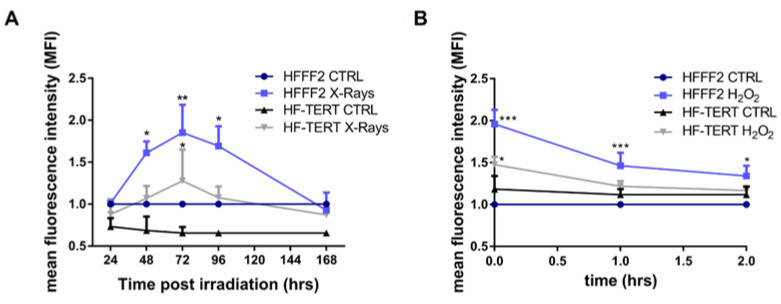
Measure of oxidative stress after X-rays irradiation or H_2_O_2_ treatment. (**A**) ROS level in control and irradiated samples for both cell lines for 168 h post-X-rays treatment. (**B**) The graph represents the comparison between both cell lines after H_2_O_2_ treatment. Measure of the level of oxidative stress in all samples was done immediately after 1 h (t0) H_2_O_2_ treatment. Successively, cells are recovered with culture media and oxidative stress is measured after 1 and 2 h (t1, t2). All results are normalized to the control values of HFFF2 cells and are expressed as mean values ± SEM of the ratio between the relative fluorescence intensity and the cell number for each day analyzed. Statistical analysis is performed between treated and control samples for both cell lines. * *p* < 0.05; ** *p* < 0.01; *** *p* < 0.001; by one-way ANOVA test with Tukey’s post-test.

Excluding the role of telomerase, in order to understand why HF-TERT cells had less genomic and telomeric damage than HFFF2, we evaluated oxidative stress (OS), induced by H_2_O_2_ or X-ray irradiation. Thus, we quantified reactive oxygen species (ROS) after both treatments. X-ray irradiation-induced oxidative stress in both cell lines, but with some dissimilarities (Figure 2A): in normal cells, OS increased after 48 h, reaching its maximum level at 72 h and subsequently decreasing up to 168 h since X-rays exposure; on the contrary, in HF-TERT cells we can note a statistically significant increase in OS only 72 h after treatment that went down immediately after 96 h. After the treatment with hydrogen peroxide for 1 h, ROS production was measured immediately (t0), after 1 h of cellular growth in culture medium (t1) and after 2 h (t2) of recovery (Figure 2B). Treated HFFF2 cells displayed a high level of ROS production immediately after treatment, which persists until t2 time. The same trend is observed in HF-TERT but with a lower level of ROS that returned at the control level after 1 h of recovery. This lower level of OS than that observed in normal fibroblasts could explain the less genomic and telomeric damage.

### 2.2. hTERT-Overexpressing Cells Display Enhanced Antioxidant Defense

**Figure 3:**
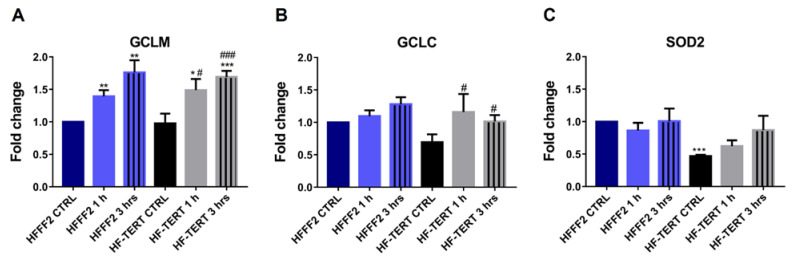
Expression level of *GCLM, GCLC* and *SOD2* genes. RT-qPCR of *GLCM* (**A**), *GCLC* (**B**) and *SOD2* (**C**) in untreated and treated normal and hTERT-overexpressing cells after 1 and 3 h of recovery. The values are normalized to the control HFFF2 cells and are expressed as mean values ± SEM. Statistical analysis (*) is performed between each sample and control HFFF2 cells (* *p* < 0.05; ** *p* < 0.01; *** *p* < 0.001; *t*-test), or between treated and untreated HF-TERT cells (# *p* < 0.05; ### *p* < 0.001; *t*-test).

Since HF-TERT cells showed a lower OS, we hypothesized that TERT could improve cellular antioxidant defense through an increase in specific factors involved in the response, such as Glutamate Cysteine Ligase (GCL) and manganese superoxide dismutase (MnSOD or SOD2) [46]. GCL is a heterodimeric protein composed of a catalytic (GCLC) and a modifier (GCLM) subunit [47], and catalyzes the formation of the cellular antioxidant glutathione (GSH). The gene expression of *GCLM, GCLC* and *SOD2* was measured by RT-qPCR in normal fibroblasts and hTERT-overexpressing cells, after 1 and 3 h of recovery following H_2_O_2_ treatment. In HFFF2 we did not observe any change in the expression of *GCLC* and *SOD2* genes while we noted an increase in *GCLM* expression after 1 h and 3 h after treatment. On the contrary, in HF-TERT cells we observed an increase in gene expression for both GCL subunits and for SOD2 after 1 and 3 h of recovery (Figure 3A–C). It is interesting to note that TERT over-expressing cells are characterized by lower basal levels of GCLC and SOD2 compared to HFFF2; this finding could be ascribable to fainter oxidative stress in HF-TERT cells, which requires lower levels of the proteins involved in the antioxidant response.

**Figure 4:**
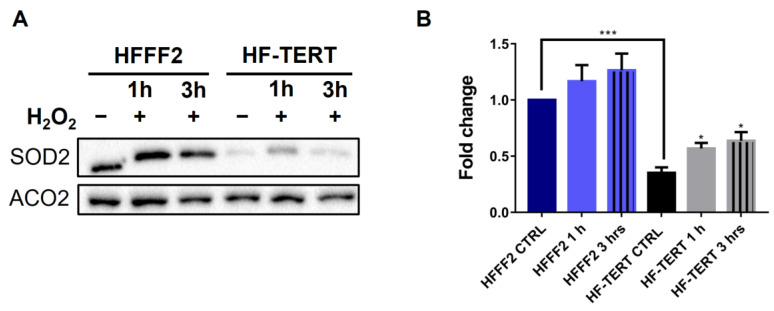
SOD2 protein level evaluated after H_2_O_2_ treatment. (**A**) Western blot of SOD2 and Aconitase 2 (ACO2, used as a control) in HFFF2 and HF-TERT cells at 1 or 3 h of recovery after H_2_O_2_ treatment. (**B**) Quantification of SOD2 protein level in both cell lines after H_2_O_2_ treatment. Data represent mean ± SEM. Statistical analysis is performed between HF-TERT and HFFF2 untreated cells, or between treated and untreated HF-TERT. * *p* < 0.05; *** *p* < 0.001 by *t*-test.

Considering that we observed *SOD2* gene modulation only in HF-TERT cells and that TERT was previously shown to induce an increase in SOD2 protein level [27], we also analyzed the protein levels of SOD2 by Western Blot analysis (Figure 4A) in both cell lines. Differently from the results on gene expression, in HFFF2 cells we noted a slight increase in SOD2 protein level after treatment. On the other hand, TERT-overexpressing cells displayed a significant increase in SOD2 protein level after hydrogen peroxide treatment, in accordance with gene expression (Figure 4B), confirming a different response to the OS of the two cell lines. As observed for gene expression, SOD2 protein levels in HF-TERT cells are lower compared to normal cells (both in untreated and treated samples), but the increase induced by H_2_O_2_ treatment is more prominent (after 3 h, 80% in HF-TERT compared to 26% in HFFF2). 

### 2.3. TERT Is Accumulated in Mitochondria under Oxidative Stress

TERT protein is characterized by a nuclear export signal, responsible for its translocation to the cytoplasm; here, it can be subjected to ubiquitination and consequent degradation or directed into mitochondria, thanks to a specific N-terminal sequence, the mitochondria transport signal (MTS) [33].

**Figure 5:**
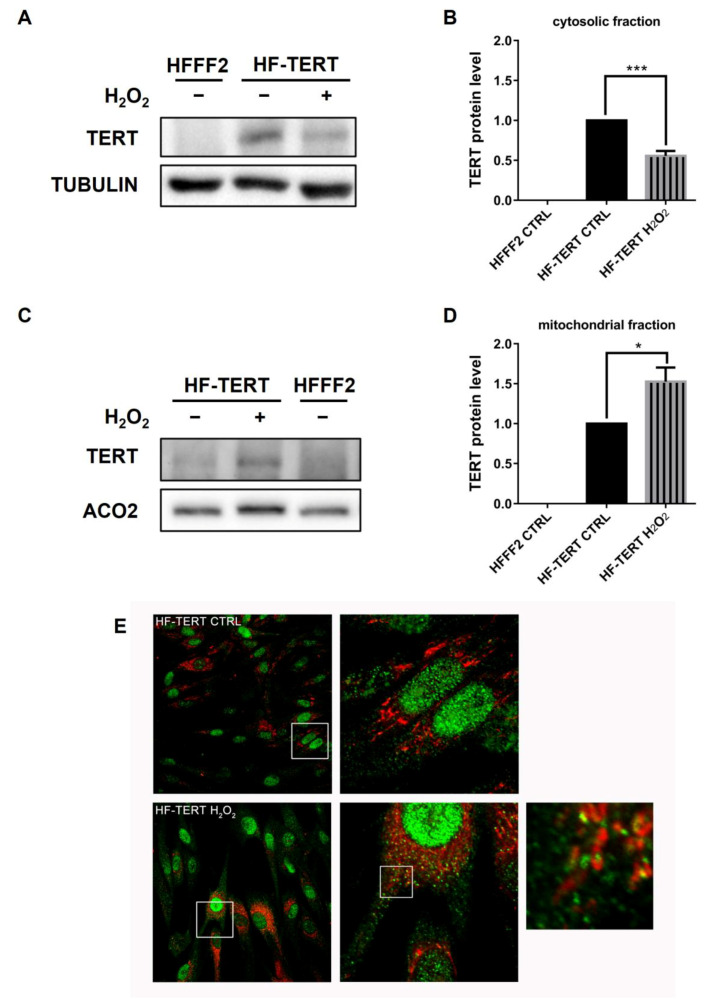
TERT localization in mitochondrial and cytosolic fraction. (**A**) Representative Western blot shows specific bands for cytosolic level of TERT and tubulin, used as control protein, in untreated and treated HFFF2 or HF-TERT cells. (**B**) Level of TERT protein in cytosol after 3 h of H_2_O_2_ treatment, normalized to tubulin signal. (**C**) Representative Western blot of mitochondrial level of TERT and ACO2, used as control protein, in untreated HFFF2 and in untreated and treated HF-TERT cells 3 h after treatment. (**D**) Quantification of mitochondrial level of TERT protein, normalized to ACO2 signal. (**E**) Image of cells stained for TERT antibody (green) and Mitospy (red) in untreated (upper panel) and treated (lower panel) HF-TERT cells. The enlarged images show localization of TERT protein into mitochondria. Data represent mean ± SEM. * *p* < 0.05; *** *p* < 0.001; by *t*-test.

TERT protein is principally localized within the nucleus, but it was also demonstrated that upon oxidative stress TERT is transported from the nucleus to the cytoplasm [32]. Thus, we analyzed the TERT level in the cytosolic fraction of untreated HFFF2 cells as well as untreated and H_2_O_2_-treated HF-TERT; we observed a decrease in the cytoplasmatic TERT amount after treatment (Figure 5A,B). Considering this result, we have wondered if the observed TERT reduction in cytosol after hydrogen peroxide treatment was caused by its degradation. Lee et al. discovered that the CHIP chaperone physically interacts with hTERT in the cytoplasm, resulting in hTERT cytoplasmatic degradation by the proteasome [48,49]. Western blot showed that CHIP expression in cytoplasm did not change after hydrogen peroxide treatment (Figure S5A,B), suggesting that the reason for TERT modulation could be due to a mechanism different from its degradation.

Since the TERT sequence is characterized by the presence of the MTS (mitochondrial targeting sequence) responsible for its mitochondrial localization [33], we supposed that the reduction in cytosolic TERT was not due to degradation but to its translocation in mitochondria. Therefore, we investigated the presence of TERT protein in the mitochondrial fraction in untreated HFFF2 cells and in untreated and H_2_O_2_-treated HF-TERT cells (Figure 5C). Western blot analysis showed an increase in TERT protein in mitochondria following treatment with hydrogen peroxide (Figure 5D). These results were also confirmed by double-fluorescent labeling with anti-TERT antibody and the mitochondrial localization probe Mitospy (Figure 5E). Untreated HF-TERT cells showed a prevalence of TERT signals in the nucleus but also a few signals in cytosol (Figure 5E, upper panel). H_2_O_2_-treated HF-TERT cells displayed a large amount of colocalization between TERT and mitochondria as shown in Figure 5E, lower panel.

Moreover, the RQ-TRAP assay performed after H_2_O_2_ treatment showed that telomerase activity is not affected by the observed different TERT localization in cellular compartments (Figure S6). 

**Figure 6:**
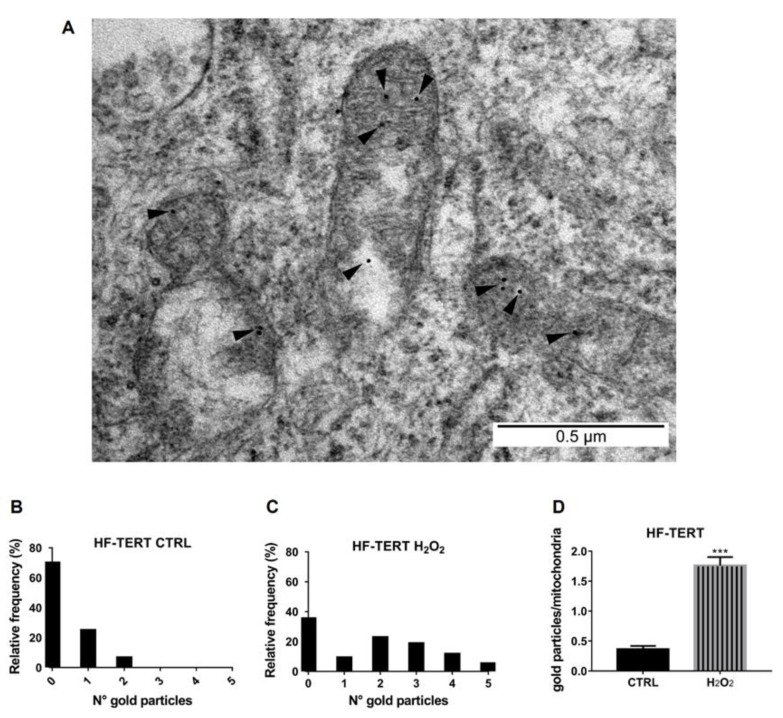
TERT detection within the mitochondria. (**A**) TEM representative micrograph of mitochondria in H_2_O_2_-treated HF-TERT cells. Magnification of TERT immunogold labeling in mitochondria (see black arrowheads). Scale bar: 0.5 µm. (**B**) Frequency of mitochondria containing a different number of gold particles in HF-TERT cells. (**C**) Frequency of mitochondria containing a different number of gold particles in HF-TERT H_2_O_2_ cells. The frequency of mitochondria scored was about 100. (**D**) Quantification of TERT particles in mitochondria. *** *p* < 0. 001; by *t*-test.

In order to assert with certainty TERT localization within mitochondria, we performed experiments with transmission electron microscopy (TEM) using immunogold particles in untreated and treated HF-TERT cells (Figure 6A). As shown in Figure 6B in HF-TERT cells, we saw most mitochondria without gold particles (mean of 69.4 mitochondria), but there were mitochondria with one or two particles (24.4 and 6.1, respectively). In HF-TERT-treated cells, the distribution of gold particles appears different (Figure 6C). We observed a decrease in mitochondria without gold particles (34.9), instead an increase in mitochondria with more than two particles (22.2 with two particles; 18.2 with three particles; 11.1 with four particles; and 4.7 with five particles). Furthermore, immunogold TEM analysis confirmed the Western blot results, and we can conclude that TERT protein was translocated into mitochondria after H_2_O_2_ treatment (Figure 6D), and this translocation does not induce nuclear telomerase activity change.

### 2.4. Effects of TERT on Mitochondrial Ultrastructural Features and Function

**Figure 7:**
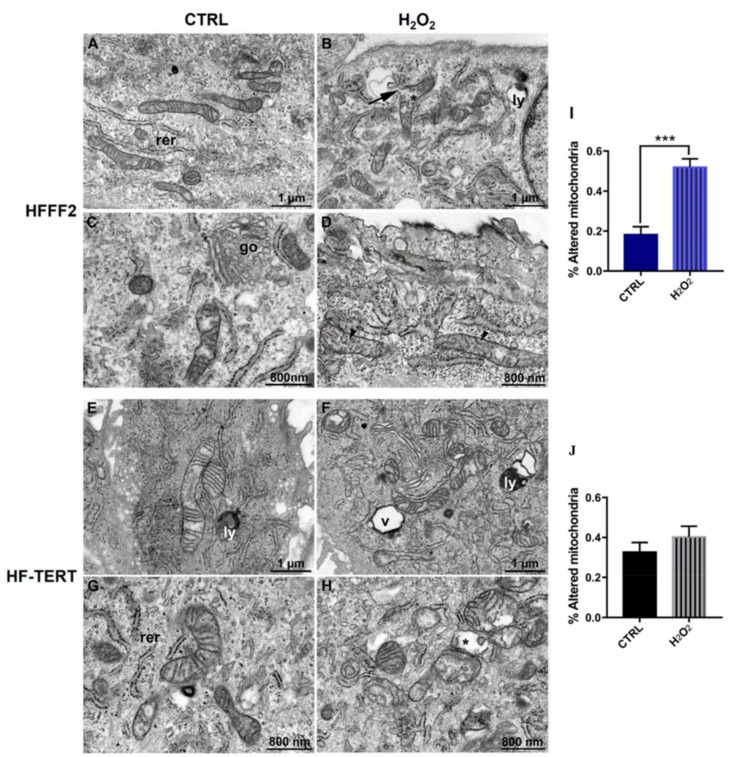
FIB/SEM micrographs illustrating mitochondrial morphology in HFFF2 (**A**–**D**) and HF-TERT (**E**–**H**) cells, treated and untreated with H_2_O_2_. In untreated cells, mitochondria display a typically elongated shape and numerous parallel-oriented *cristae*. Following treatment, mitochondrial swelling (asterisks), with matrix expansion and *cristae* dysmorphic features, including numerical reduction, spatial disorganization and/or fragmentation (arrowheads) are observed. Rupture of the outer membrane is occasionally found (arrow). The diagrams report mitochondria alteration in untreated and treated (**I**) HFFF2 cells and (**J**) HF-TERT cells after 3-hour recovery. Data represent mean ± SEM. *** *p* < 0.001; by Student’s *t*-test. Acronyms: (go) Golgi complex, (ly) lysosome, (rer) rough endoplasmic reticulum, (v) vacuole.

Ultrastructural analysis was performed by Focused Ion Beam/Scanning Electron Microscopy (FIB/SEM), to investigate overall cellular morphology and particularly, mitochondrial features. Both cell lines displayed regular cell shape and electron density, with a well-preserved plasma membrane, nuclear morphology and cytoplasmic organelles (Figure 7A–E). Mitochondria of either HFFF2 or HF-TERT cells showed consistent ultrastructural features, i.e., a typically elongated morphology with cristae arranged in a parallel manner and a moderate electron-dense matrix (Figure 7C–G).

Following H_2_O_2_ treatment, the general morphological features of either cell lines appeared impaired (Figure 7B–F). Numerous mitochondria showed abnormal outer and inner membrane (IMM and OMM) ultrastructure, with pronounced swelling and consequent expansion of the electron-lucent matrix. *Cristae*, particularly, were reduced in number and lost their normal topology, being fragmented and irregularly arranged (Figure 7D–H). Occasional rupture of the OMM was also observed (Figure S7). Despite the qualitative similarity of alterations caused by oxidative insult, hTERT-overexpressing cells responded differently, in terms of mitochondrial damage extent and severity. Indeed, quantitative analysis of randomly acquired FIB/SEM microphotographs (see M&M, for details) from longitudinally sectioned cells (Figures S8 and S9), showed a relatively small increase in abnormal mitochondria in HF-TERT cells. A highly significant difference in the number of damaged mitochondria was found in HFFF2 cells after treatment, while in HF-TERT cells this difference fails to reach significance (diagrams in Figure 7). Moreover, numerous lysosomes and autophagic vesicles were observed after treatment, especially in TERT-overexpressing cells.

**Figure 8:**
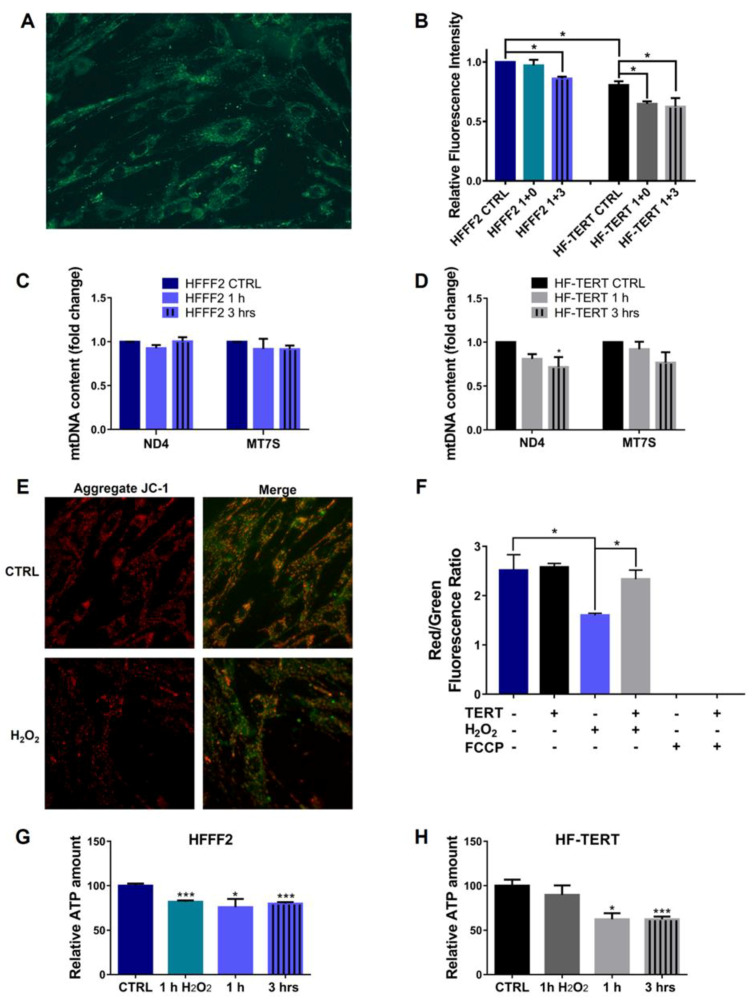
Mitochondrial function and amount in untreated and treated HFFF2 and HF-TERT. (**A**) Representative image of HFFF2 cells stained with MitoTracker Green. (**B**) Mitochondrial mass in untreated and treated HFFF2 cells and HF-TERT cells immediately after 1 h, after 1 and 3 h of recovery from H_2_O_2_ treatment. (**C**,**D**) qPCR for ND4 and MT-7S mitochondrial genes in untreated and treated HFFF2 (**C**) and HF-TERT cells (**D**) after 1 and 3 h of recovery. (**E**) Mitochondrial membrane potential (ΔΨ_m_) measured by JC-1, red to green fluorescence intensity ratio. (**F**) Quantification data of JC-1 after 1 h of H_2_O_2_. FCCP (10 μM) is used as the positive control. ATP level in untreated and treated HFFF2 (**G**) and HF-TERT (**H**) cells. All values are expressed as mean values ± SEM. Statistical analysis is performed between treated and control samples of both cells. * *p* < 0.05; *** *p* < 0.001; by *t*-test.

Successively, we analyzed the mitochondrial mass to determine if there were changes in metabolic functions afterwards hydrogen peroxide treatment, by using Mitotracker Green, as shown in Figure 8A,B. In normal cells, we did not observe any change immediately after treatment, but we saw a slight decrease in mitochondrial mass after 3 h of recovery. On the contrary, untreated HF-TERT cells displayed less mitochondrial mass compared to their untreated counterpart. After H_2_O_2_ treatment, mitochondrial mass was already significantly reduced immediately and after 3 h of recovery. On the other hand, mtDNA copy number was evaluated by quantification of the MT-ND4 gene and MT-7SDNA in HFFF2 and HF-TERT cells after treatment (Figure 8C,D). qPCR results showed a slight reduction in the mtDNA copy number in HF-TERT after hydrogen peroxide treatment, while in normal cells, the mtDNA copy number was unaffected after H_2_O_2_ treatment.

To evaluate the mitochondrial function after TERT translocation, we first measured the mitochondrial membrane potential (Δψ_m_) using the fluorescent dye JC-1, a molecule which accumulates in mitochondria in a membrane potential-dependent manner, forming reversible aggregates (red emission) (Figure 8E). Following mitochondria depolarization JC-1 shifts to a monomeric form resulting in green emission [50]. HFFF2 cells exposed to H_2_O_2_ for 1 h showed a significant decrease in the red to green fluorescence intensity ratio respect to control cells, indicating a mitochondrial depolarization. Differently, H_2_O_2_-treatment of HF-TERT cells did not induce any mitochondrial depolarization as observed by no JC-1 ratio change (Figure 8F). Interestingly, the J-aggregates in treated normal cells were significantly less abundant than treated hTERT-overexpressing cells. In addition, we measured the ATP levels, the source of energy within the cell produced by mitochondria [51]. After H_2_O_2_ treatment, HFFF2 cells displayed a significant decrease in ATP level for all the analyzed timepoints (immediately, after 1 h and after 3 h of recovery), while HF-TERT cells showed ATP reduction only after 1 or 3 h of recovery (Figure 8G,H). 

### 2.5. TFAM Expression Increases in HF-TERT Cells after Treatment

**Figure 9:**
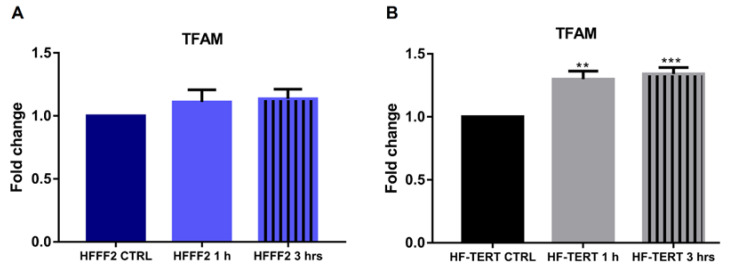
Expression level of TFAM gene in untreated and treated HFFF2 (**A**) and HF-TERT (**B**) after H_2_O_2_ treatment. The values are expressed as mean values ± SEM. Statistical analysis is performed between treated and control samples. ** *p* < 0.01; *** *p* < 0.001; by *t*-test.

Mitochondrial transcription factor A (TFAM) is a member of the high-mobility group (HMG) [52], able to directly bind mtDNA, but it is also involved in many functions such as helping mitochondrial DNA in transcription and replication [53,54]. As a possible indication of the modulation of this process, we analyzed the expression of the *TFAM* gene in HFFF2 and HF-TERT cells at 1 and 3 h after H_2_O_2_ treatment. Even though in normal fibroblasts we observed a slight not significant increase in the TFAM expression after treatment (Figure 9A), only in HF-TERT cells, TFAM gene showed a significantly increased expression at both times analyzed (Figure 9B) suggesting likely different regulation of mtDNA functions. 

## 3. Discussion

TERT, together with TERC, aside from the well-established function in telomere lengthening, has non-canonical functions as a transcriptional regulator of genes in different pathways but also has an anti-apoptotic and antioxidant role [24,26,27]. In recent years, many different reports have focused on the role of TERT in increasing resistance to specific DNA-damaging agents and in reducing cellular ROS levels, with a protective effect on the cellular redox status [26,30].

Taking advantage of normal primary fibroblasts (HFFF2), cells without any mutation and that lack TERT expression and, consequently, telomerase activity, transduction with *hTERT* gene allowed us to study TERT telomere-independent functions after oxidative stress (OS) damage, induced by physical and chemical agents (X-rays and H_2_O_2_).

Firstly, we evaluated total and telomeric DNA damage induction using a phosphorylated form of histone H2AX (γH2AX), recruited after DSB [38]. Both genotoxic agents induced a significant increase in DNA damage. However, HFFF2 cells had a greater damage induction that persisted for many days after treatment, while TERT-overexpressing fibroblasts had less DNA damage, which was completely repaired in the following days.

The same results have been observed in telomeric sequences. Telomeric DNA is a preferential target of OS due to a high content of guanine residues, which makes it susceptible to oxidative damage. In addition, the telomeric region is also less efficiently repaired than other parts of the genome [40,55]. In this case, we have analyzed the colocalization of γH2AX and TRF1, a specific telomeric protein, in order to analyze Telomere Induced Dysfunctional Foci (TIFs) and we have evidenced different levels of TIFs induction between the two cell lines. Normal cells showed higher telomeric damage after X-rays and H_2_O_2_ treatment compared to HF-TERT, confirming results obtained from the analysis of genomic DNA damage. These observations are consistent with the results reported by Sharma and coll. that demonstrated a better response to DNA damage in cells with ectopic expression of TERT, after treatment with ionizing radiation and cisplatin [30]. In addition, in cells with suppression of TERT, an increase in radiosensitivity, diminished capacity for DNA repair and fragmented chromosomes were observed, demonstrating that loss of TERT impairs the DNA-damage response [56]. The lower induction of genomic and telomeric damage observed by us and other authors in normal cells overexpressing TERT, could, however, also result from protective effects of TERT on cellular redox status [30,36]. Following this hypothesis, we decide to assess levels of OS, induced both by H_2_O_2_ or by X-ray irradiation, considering that also X-rays are able to induce oxidative stress [45]. Thus, we have quantified reactive oxygen species (ROS) after both treatments. Although the two cell lines displayed a similar trend, HF-TERT cells seemed to have less induction of OS, both after X-ray irradiation and H_2_O_2_ treatment. These results are similar to those of Ahmed et al. (2008), in which over-expressing hTERT fibroblasts were shown to display reduced levels of intracellular ROS and therefore reduced oxidative stress under both basal conditions and induced chronic oxidative stress [36]. This evidence was also observed in non-transformed cells, as reported by Yang and colleagues [22]. The authors using embryonic stem cells showed ROS levels lower than that observed after the TERT knockdown, suggesting resistance to oxidative stress. In tumor cells, Indran et al., 2011, have demonstrated that TERT reduces basal cellular ROS level and intracellular ROS in response to different stimuli. *Vice versa*, TERT downregulation in the same cells induces an increase in ROS. These anti-oxidative effects of TERT are correlated to an increase in Glutathione (GSH) and non-oxidized peroxiredoxin [26]. 

In agreement with different authors, our results indicate less induction of ROS in HF-TERT than in normal fibroblasts; thus, we have investigated different antioxidant genes such as Glutamate Cysteine Ligase (GCL) and manganese superoxide dismutase (MnSOD or SOD2) [27,57]. GCL is a heterodimeric protein, composed of a catalytic (GCLC) and a modifier (GCLM) subunit [47], which catalyzes the formation of the cellular antioxidant glutathione (GSH). While GCLM increased in both cell lines, GCLC increased only in HF-TERT cells. This increase probably leads to a greater GCL heterodimer formation and consequent increase in antioxidant ability. We also observed increases in SOD2 mRNA and protein levels in HF-TERT cells after treatment, confirming a better antioxidant response compared to normal fibroblasts. Collectively, these observations are in agreement with the ability of hTERT (via the interaction with NF-kB) to induce the expression of antioxidant genes [58,59,60]. Moreover, it is interesting to note that TERT antioxidant activity is present even at basal conditions, as revealed by the observation of a lower level of ROS and of some factors involved in the cellular antioxidant defense (GCL, SOD2).

Interestingly, in recent years, it was found that TERT functions are not limited to the nucleus. Haendeler and colleagues have discovered that the Scr kinase family regulates TERT export from the nucleus to the cytoplasm under oxidative stress [32]. ROS provokes rapid activation of the Src kinase, which induces phosphorylation of nuclear TERT on tyrosine 707. This phosphorylated form interacts with the nuclear export receptor CRM1/exportin, and so, it is actively transported through nuclear pores [32,61]. This result clearly identifies oxidative stress as the main trigger for TERT nuclear exclusion. Due to this interesting data and with the aim of studying TERT extra telomeric roles, in our model, we analyzed TERT level in the cytosolic fraction after hydrogen peroxide treatment and we observed a decrease in TERT amount in H_2_O_2_-treated HF-TERT cells. Firstly, we considered the hypothesis that this reduction could be due to TERT ubiquitination and consequent degradation, induced by the interaction with the chaperone CHIP [48,49]. We investigated if CHIP protein could be responsible for the observed TERT reduction. However, our results showed that the CHIP level in cytosol did not change after treatment. Thus, the reduction in TERT level seemed to be not ascribable to an increased degradation but probably to its translocation in another compartment. In fact, Santos and co-authors have identified a specific N-terminal sequence of TERT, the mitochondria transport signal (MTS), that allows TERT to be transported into mitochondria through the mitochondrial membrane [33]. Haendeler and co-workers have found that TERT interacts with both TOM20 and TOM40 at the mitochondrial outer membrane and TIM23 at the inner membrane and TERT resides in the mitochondrial matrix [34]. Different authors in recent years have confirmed the presence of TERT in mitochondria, using different cellular models, and have shown that hydrogen peroxide treatment is able to increase its translocation into mitochondria [34,36,37]. Using our cellular models and different techniques we have demonstrated that TERT is located in mitochondria and is further translocated inside the organelle after hydrogen peroxide treatment. Treated HF-TERT cells showed an increased amount of TERT into mitochondria, compared to untreated cells, indicating that oxidative stress could represent a trigger for TERT translocation into mitochondria. We have demonstrated the presence of TERT protein in the mitochondrial fraction by Western blot, which increased after H_2_O_2_ treatment. Subsequently, both immunofluorescence and transmission electron microscopy (TEM) have corroborated previously observed results. To date, various methods have been used to reveal the localization of TERT in mitochondria, including immunoblotting, coimmunoprecipitation and immunofluorescence [33,34,36,62]; however, for the first time in this paper, TERT was detected inside mitochondria by TEM immunogold labeling, which is one of the most sensitive methods for localization and quantification of antigens in different cellular compartments or organelles [63,64].

After showing that, in our conditions, TERT moves into mitochondria after oxidative stress, our attention was focused on the study of the possible effects of its translocation, probably influencing mitochondrial status and functionality. Thus, we tested the mitochondrial function after H_2_O_2_ treatment evaluating the mitochondrial membrane potential (Δψ_m_) and we showed mitochondrial depolarization only in treated normal cells. Differently, in HF-TERT cells we did not observe any change in Δψ_m_ level, suggesting that TERT probably ameliorates mitochondrial health status. Successively, in transduced fibroblasts we have observed a reduction in mitochondrial mass and ATP after H_2_O_2_-treatment, consequently accompanied by a reduction in mtDNA. On the other hand, ultrastructural analyses showed in H_2_O_2_-treated HF-TERT cells fewer mitochondria abnormalities compared to normal cells. Taking into account all the mitochondrial data, HF-TERT fibroblasts contained a lower number of mitochondria (observed as mass) but are characterized by a better function and morphology compared to normal fibroblasts (see in Δψ_m_ and ultrastructural analysis). Furthermore, data obtained from the analysis of TFAM, typically involved in mtDNA transcription and replication, indicated only in HF-TERT an increase in TFAM expression suggesting that under OS, TERT induces TFAM as an early step of a mitochondrial renewal process. Therefore, we can speculate that if on one hand TERT could activate a mechanism to discard damaged mitochondria; on the other hand, this protein may be responsible for the restoration of new healthy mitochondria, as suggested by TFAM induction in TERT-overexpressing cells. This peculiar behavior of TERT, under oxidative stress conditions, could reveal further important roles of this protein, that seem to be apparently contradictory but anyway dedicated to guarantee cellular survival. It will be interesting deeper investigate this hypothesis, in order to clarify which are the specific actors involved in mitochondria biogenesis and in mitochondria disruption. 

In conclusion, the use of human primary fibroblasts and their transduced counterpart allowed us to study the role of TERT in normal cells, in a normal cellular context, free of any gene mutation. Our results have confirmed previous literature data, highlighting even more the antioxidant role of TERT, both under basal and stress conditions. In fact, TERT untreated cells displayed a better response than normal primary fibroblasts for many parameters. Most importantly, we have demonstrated TERT translocation into mitochondria induced by oxidative stress; this translocation seems to be related to the TERT protective role in preserving mitochondria functionality, as demonstrated by our functional and structural results. Further investigations are needed to understand the reason of TERT translocation into mitochondria induced by oxidative stress and to unravel any targets and the putative mechanism of action inside the mitochondrion.

## 4. Materials and Methods

### 4.1. Cells, Culture Condition and Retroviral Transduction

Human Fetal Foreskin Fibroblasts (HFFF2) (ECACC, Salisbury, UK) and Human Epithelial Kidney cells (HEK 293) were grown in D-MEM. Cell medium was supplemented with 10% fetal bovine serum (FBS), 10,000 units/mL penicillin, 10 mg/mL streptomycin and 2 mM L-glutamine (Euroclone, Milan, Italy). All cell lines were maintained in a humidified incubator at 37 °C, with 95% relative humidity and 5% CO_2_. 

As described by Counter et al. [65], pBabe-Puro-hTERT (Addgene plasmid #1771) vector plasmid containing the cDNA of hTERT protein was transfected into HEK 293 using Lipofectamine 2000 (Thermo Fisher Scientific, USA) and Ampho Retrovirus Packaging Vector (Novus Biological, Englewood, CO, USA) to obtain retroviral vector particles. Four days later, the supernatant from these cells were used to infect HFFF2 cells to establish the HF-TERT cell line. After the infection, cells were selected with puromycin (2 μg/mL) (Tocris, Ellisville, MO, USA) for 5 days. Human glioblastoma astrocytomas (U251MG) were grown in MEM, supplemented with 10% fetal bovine serum (FBS), 10,000 units/ml penicillin, 10 mg/ml streptomycin, 2 mM L-glutamine, 1% Non-Essential Amino Acids and 1 mM Sodium Pyruvate (Euroclone, Milano, Italy). U251 were used in RQ-TRAP assay as positive control of telomerase activity.

### 4.2. Real-time Quantitative—Telomeric Repeat Amplification Protocol Assay (RQ-TRAP)

Telomerase activity was measured by the SYBR green RT-qPCR assay, which was conducted as described elsewhere [66] with minor modifications. Briefly, the reaction was performed with protein extracts, 0.1 µg of telomerase primer TS, and 0.05 µg of anchored return primer ACX, in SYBR Green PCR Master Mix (Biorad, Hercules, CA, USA). The primer sequences were those reported by Kim and Wu [67]. The reaction was performed using the Agilent AriaMax real-time PCR system (Agilent Technologies, Santa Clara, CA, USA), samples were incubated for 30 min at 30 °C and amplified in 40 PCR cycles with 30 s at 95 °C and 90 s at 62 °C (two step PCR). HF-TERT heat-treated cells samples were obtained by boiling protein extract at 85 °C for 10 min. Telomerase activity was expressed relative to the telomerase positive (U251MG) cells and HF-TERT heat treated cell was used as negative control. Each sample was analyzed in triplicate in at least three independent experiments.

### 4.3. H_2_O_2_ and X-rays Treatment

Cells were subjected alternatively to two different genotoxic agents, X-ray irradiation or hydrogen peroxide. In the first case, cells were X-rays irradiated at room temperature using a Gilardoni apparatus (200 kV, 6 mA, dose-rate 0.51 Gy/min) with a dose of 4 Gy, then were seeded at the requested density in fresh medium. Unirradiated cells were used as the control in all the experiments and were seeded at the requested density as irradiated cells. 

In the case of H_2_O_2_ treatment, cells were treated with H_2_O_2_, 24 h after seeding in a complete medium, for 1 h at 37 °C in incubator at the final concentration of 200 μM. After 1 h of treatment, H_2_O_2_ was removed and cells were grown in complete medium for 1 or 3 h of recovery. Cells were examined at different times after treatment and were compared to parallel cultured control HFFF2 cells grown in the medium without H_2_O_2_.

### 4.4. Growth Curves

After treatment, 1 × 10^5^ cells were seeded in a Petri dish and every 24 h were detached and counted up to 120 h. All data points were performed at least in three different experiments.

### 4.5. Genomic and Telomeric γH2AX Immunofluorescence Staining

After treatment, the cells were fixed with 4% paraformaldehyde (Sigma Aldrich‚ Burlington, MA, USA), permeabilized in Triton X-100 and blocked with 1% bovine serum albumin (BSA). Slides were incubated with a mouse monoclonal anti-phospho-histone H2AX antibody (Millipore, Burlington, MA, USA) in combination, when needed, with rabbit telomeric protein TRF1 antibody (Santa Cruz Biotechnology, Dallas, TX, USA), overnight at 4 °C. Samples were washed in PBS then exposed to the secondary anti-mouse Alexa 546 antibody (Invitrogen, Waltham, MA, USA) and anti-rabbit Alexa 488 (Invitrogen, Waltham, MA, USA) for 1 h. DNA was counterstained by DAPI in Vectashield (Vector Laboratories Inc, Newark, CA, USA). Slides were analyzed using Axio Imager M1 microscope (Carl Zeiss) equipped with a CCD camera. The frequency of foci per cell were scored in 50 nuclei in at least three independent experiments.

### 4.6. Collection of Chromosome Spreads and Quantitative-Fluorescence “in situ” Hybridization Analysis (Q-FISH)

After treatment, chromosome spreads were obtained following 30 min incubation in 30 μM calyculin-A (Wako, Germany) [68]. Spreads of these prematurely condensed chromosomes (PCC) were prepared by a standard procedure, consisting of treatment with a hypotonic solution (75 mM KCl) followed by fixation in freshly prepared Carnoy solution. Q-FISH staining was performed as described by Berardinelli et al. [69] with minor modifications. Briefly slides and probes (Cy3 linked telomeric and chromosome 2 centromeric Peptide Nucleic Acid PNA probes; PANAGENE, Republic of Korea) were co-denatured at 80 °C and hybridized for 2 h at room temperature in a humidified chamber. Slides were counterstained with DAPI in Vectashield. Images were captured at a 63× magnification with Axio Imager M1 microscope equipped with a CCD camera. The telomere size was analyzed with ISIS software (MetaSystems, Altlußheim, Germany). In particular, the software calculates telomere lengths as the ratio between the total telomeres fluorescence (T) and the fluorescence of the centromere of the two chromosomes 2 (C), thus data were expressed as a percentage (T/C%). Experiments were repeated at least three times, and at least 10 metaphases were scored for each experiment.

### 4.7. Intracellular Reactive Oxygen Species (ROS) Determination

The accumulation of intracellular ROS level was detected after irradiation or hydrogen peroxide treatment, with the ROS detection assay kit purchased from BioVision. Cells were irradiated and analyzed at different times. After irradiation 1 × 10^4^ cells were seeded inside 96-multiwell plates. For hydrogen peroxide treatment, 2 × 10^5^ cells were seeded in a Petri dish. The culture medium was discarded, new medium containing the fluorogenic probe H2DCFDA was added (1× ROS label solution, as indicated in the manufacturer’s protocol) and incubated for 30 min at 37 °C. Samples were treated with 200 µM H_2_O_2_ for 1 h. Subsequently, hydrogen peroxide was removed and added in a complete medium. In the untreated samples, after probe incubation, were added culture media. At the end of stimulation, cells were analyzed in a FL-1 fluorescence channel using a CytoFlex (Beckman Coulter, Pasadena, CA, USA) flow cytometer. About 20,000 events/samples were analyzed for each condition. Analysis was performed with a CytExpert v2.2 software (Beckman Coulter, Brea, CA, USA). Doublet discrimination was performed by an electronic gate on FSC-Area vs. FSC-Height. Experiments were repeated at least three times.

### 4.8. Analysis of Biogenesis and Glutathione Genes Expression

Total RNA was extracted from HFFF2 and HF-TERT cells using the Total RNA Purification Plus Kit (Norgen Biotek Corp, Thorold, ON, Canada) and has been reverse-transcribed using a LunaScript™ RT SuperMix Kit (New England Biolabs, Ipswich. MA, USA). Quantitative reverse transcription PCR (RT-qPCR) analysis has been performed using the TaqMan Universal PCR Master Mix (Applied Biosystems, Paisley, UK), TFAM (Hs00377764_g1), GCLC (Hs00155249_m1), GCLM (Hs00978072_m1) and SOD2 (Hs00167309_m1) have been used and the relative abundance of each target transcript has been normalized to the expression level of GAPDH (Hs99999905_m1). Data have been analyzed using the 2^−ΔΔCt^ method and reported as fold change relative to controls (untreated cells). Experiments were repeated at least three times.

### 4.9. Protein Extraction from Whole Cell and Subcellular Fractions

Cells were treated as described above and lysed in 20 mM Tris HCl pH 7.5, 150 mM NaCl, 1 mM EDTA, 1% Triton-100X and protease inhibitors, to obtain the whole-cell extract. Protein extracts of mitochondrial and cytosolic fractions were isolated using the Mitochondria/Cytosol Fractionation Kit (Abcam, Waltham, MA, USA) and according to the manufacturer’s protocol.

### 4.10. Western Blotting

Protein extracts (50 μg) were loaded on an SDS-PAGE and transferred onto a polyvinylidene fluoride (PVDF) membrane (Immobilion-P, Millipore). After blocking in 3% BSA, membranes were incubated with the following primary antibodies: anti-β-Actin (C4) (sc-47778, Santa Cruz Biotechnology, Dallas, TX, USA), anti-TERT (600-401-252S, Rockland), anti-SOD2 (sc-137254, Santa Cruz Biotechnology), anti-Aco2 (NBP1-32781, Novus Biological), anti-CHIP (ab2917, Abcam), anti-COX IV (ab14744, Abcam) and anti-α-tubulin (T6199, Sigma-Aldrich). Finally, membranes were incubated with the appropriate HRP-conjugated secondary antibody (Bio-Rad Laboratories, Hercules, CA, USA). Proteins were visualized using Clarity^TM^ Western ECL substrates (Bio-Rad). Images were acquired on ChemiDoc™ Imaging system (Bio-Rad) and protein levels were quantified using the Image Lab software (Bio-Rad). Experiments were repeated at least three times.

### 4.11. Immunofluorescence Co-Staining of Mitochondria

Cells were seeded on chambered coverslips (μ-Slide 8 well, Ibidi) and subjected to H_2_O_2_ treatment as described above, and after 3 h of recovery, cells were incubated with 500 nM MitoSpy^TM^ (BioLegend) at 37 °C for 30 min. Then, cells were fixed with 4% paraformaldehyde (Sigma Aldrich) for 15 min, permeabilized and blocked in 5% BSA for 1 h. Slides were incubated with a rabbit polyclonal anti-TERT (Rockland) overnight at 4 °C and then exposed to the secondary antibody (anti-rabbit Alexa 488, Invitrogen) for 1 h at 37 °C. Finally, fluorescent images were registered with Leica TCS SP5 confocal microscope and processed with LAS AF software (version 1.6.3, Leica Microsystems CMS GmbH).

### 4.12. Immunogold at TEM

Cells were centrifuged at 1800 rpm for 10 min and the resultant pellets were fixed in 4% glutaraldehyde for 2 h at 4 °C. After several washes in 0.1 M cacodylate buffer (pH 7.4), samples were post-fixed in 1% osmium tetroxide for 20 min in the dark, dehydrated in ethanol series and embedded in an Epon-Araldite 812 mixture (Sigma-Aldrich, Milan, Italy). Ultrathin sections (70 nm in thickness) were obtained with a Reichert Ultracut S ultratome (Leica, Wien, Austria) and collected on gold grids (300 mesh). After etching with 3% NaOH in methanol, slides were incubated for 30 min in a blocking solution containing PBS, 1% BSA and 0.1% Tween and then, with the rabbit antibody, anti-hTERT (Rockland) was diluted at 1:200 in BSA blocking solution for 1 h. After several washings with PBS, the primary antibody was visualized by immunostaining with the secondary goat anti-rabbit IgG (H + L)-gold conjugate antibody (GE Healthcare, Amersham, UK; particle size, 10 nm) diluted at 1:50 in BSA blocking solution for 45 min. Subsequently, slides were treated for 5 min with PBS containing 0.5% glutaraldehyde, counterstained with uranyl acetate and observed under a Jeol 1010 EX transmission electron microscope (Jeol, Tokyo, Japan). Data were recorded with a MORADA digital camera system (Olympus, Tokyo, Japan) and the frequency of mitochondria scored 100 in cells.

### 4.13. Detection of Mitochondrial Membrane Potential (ΔΨ_m_)

The fluorescent dye JC-1 (5,5′,6,6′-Tetrachloro-1,1′,3,3′-tetraethylbenzimidazolylcarbocyanine Iodide, AdipoGen) was used to assess the mitochondrial membrane potential. JC-1 is a lipophilic cation dye that exhibits a potential-dependent accumulation in mitochondria indicated by a fluorescence emission shift from green (529 nm) to red (590 nm) [50]. Cells were seeded in a Petri dish and treated with 200 µM of H_2_O_2_ for 1 h; subsequently, hydrogen peroxide was removed, cells were washed once with PBS and then incubated with 5 μg/mL JC-1, at 37 °C for 15 min. After two washes with PBS, cells were covered with fresh PBS and their fluorescence was analyzed using a Zeiss Axiophot (Carl Zeiss) fluorescent microscope. Mitochondrial uncoupler FCCP (Carbonyl cyanide-p-trifluoromethoxyphenylhydrazone, Sigma) was used as a positive control. 

Acquired images were analyzed with ImageJ (NIH, Bethesda, MD, USA) for green and red fluorescence and results were given as red/green fluorescence ratio. At least 100 cells per sample were analyzed.

### 4.14. ATP Content

The cellular ATP content was measured using a luminometric assay (ATPLITE 1 STEP, PerkinElmer, Waltham, MA, USA) as follows: 1 × 10^4^ cells per well were seeded in triplicate on 96-well microtiter. The following day cells were treated with 200 µM of H_2_O_2_ for 1 h. Subsequently, ATP measurement has been performed using the EnSpire^®^ Multimode Plate Readers. Experiments were repeated at least three times.

### 4.15. mtDNA Copy Number and Mitochondrial Mass

DNA was extracted from cells using the NucleoSpin Tissue kit for DNA (Macherey-Nagel). A total of 30 ng of DNA were used for qPCR analysis of mitochondrial DNA using a specific Taqman probe for MT-ND4 and MT-7S genes, while nuclear gene RNase P (Copy Number Reference Assay, human, RNaseP) was quantified for normalization. Data were analyzed using the 2^−ΔΔCt^ method and reported as a fold change relative to controls. Experiments were repeated at least three times.

Mitochondrial mass per cell was measured using MitoTracker Green FM (Molecular Probes, Eugene, OR, USA). A total of 1 × 10^5^ cells were seeded in a Petri dish and treated with 200 µM H_2_O_2_ for 1 h; subsequently, hydrogen peroxide was removed, cells were washed once with PBS and then incubated with 50 nM MitoTracker^TM^ Green FM (ThermoFisher, Waltham, MA, USA) for 30 min at 37 °C in the dark. After two washes with PBS, fluorescent images were acquired by Axio Imager M1 microscope (Carl Zeiss) equipped with a CCD camera. Acquired images were analyzed with ImageJ (NIH, Bethesda, MD, USA) for green fluorescence. At least 100 cells per sample were analyzed.

### 4.16. Ultrastructural Analysis

Cells were grown on glass coverslips and processed for electron microscopy, as previously described [70]. Briefly, samples were fixed with 2% formaldehyde (from paraformaldehyde) and 1% glutaraldehyde in 0.1 M cacodylate buffer, pH 7.4, at 4 °C. The subsequent steps, including osmium post-fixation, UranyLess (Electron Microscopy Science, Foster City, CA, USA) contrast and ethanol dehydration, were performed on ice, in the dark. Complete dehydration in 100% ethanol and the subsequent steps were carried out at room temperature. Cells were infiltrated with a 1:1 mixture of anhydrous ethanol and epoxy embedding medium (Sigma-Aldrich™, Cat# 45359-1EA-F, Burlington, MA, USA), then in pure resin for 90 min. The excess resin was gently removed, prior to polymerization at 60 °C for 72 h. This delicate procedure allowed us to readily identify the cell boundaries at FIB/SEM, facilitating the milling process and the cross-sectioning of the sample. Resin-embedded coverslips were secured to stubs using an adhesive carbon disc and made conductive with a thin layer of gold by a K550 sputter coater (Emithech, Kent, UK). The resulting samples were analyzed with a Dual Beam (FIB/SEM) Helios Nanolab 600 (FEI Company, Hillsboro, OR, USA) at the electron microscopy interdepartmental facility (LIME), at Roma Tre University. The cells were longitudinally cut by the ion beam operated at a voltage of 30 KV and a current of 6.5 nA. The resulting cross-sections were examined by the SEM column, scanning the backscattered electrons using a 2 KV voltage and a current of 0.17 nA.

## Data Availability

All data are available in the main text and in Supplementary Materials.

## Supplementary Materials

The following supporting information can be downloaded at: https://www.mdpi.com/article/10.3390/ijms24054450/s1.
