# Supplementary material for: TERT Extra-Telomeric Roles: Antioxidant Activity and Mitochondrial Protection
Source: Int J Mol Sci. 2023 Feb 23;24(5):4450. doi: 10.3390/ijms24054450 (PMC10002448; doi:10.3390/ijms24054450)
Supplement: Supplementary file 1 [file ijms-24-04450-s001.zip › ijms-2104034-supplementary.pdf]

Supplementary Materials

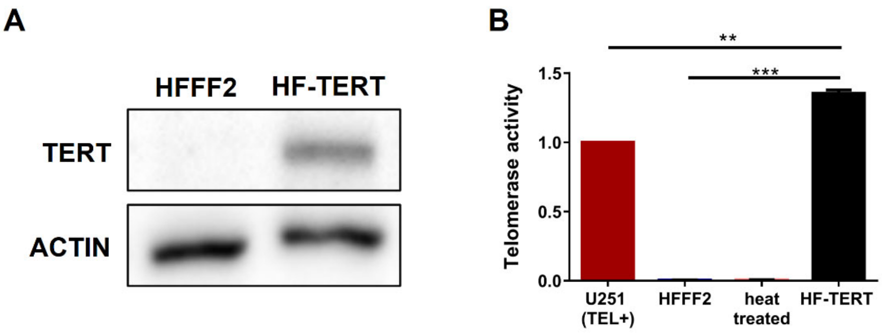

Figure S1. Efficiency of hTERT transduction in HFFF2 cell lines. (**A**) Western blot of total cellular proteins from HFFF2 and HF-TERT analyzed using anti-TERT antibody (upper panel) as well as anti-actin antibody (lower panel). (**B**) Telomerase activity measured by RQ-TRAP assay in HF-TERT fibroblasts compared to their normal counterparts and telomerase tumor cell lines (U251). U251 are used as positive control and heat-treated HF-TERT and HFFF2 cell lines are used as negative control. The error bars indicate the mean ± SEM. Statistical analysis is performed both between normal and transduced cells and between U251 and transduced cells. ** $p < 0.01$; *** $p < 0.001$ by *t*-test.

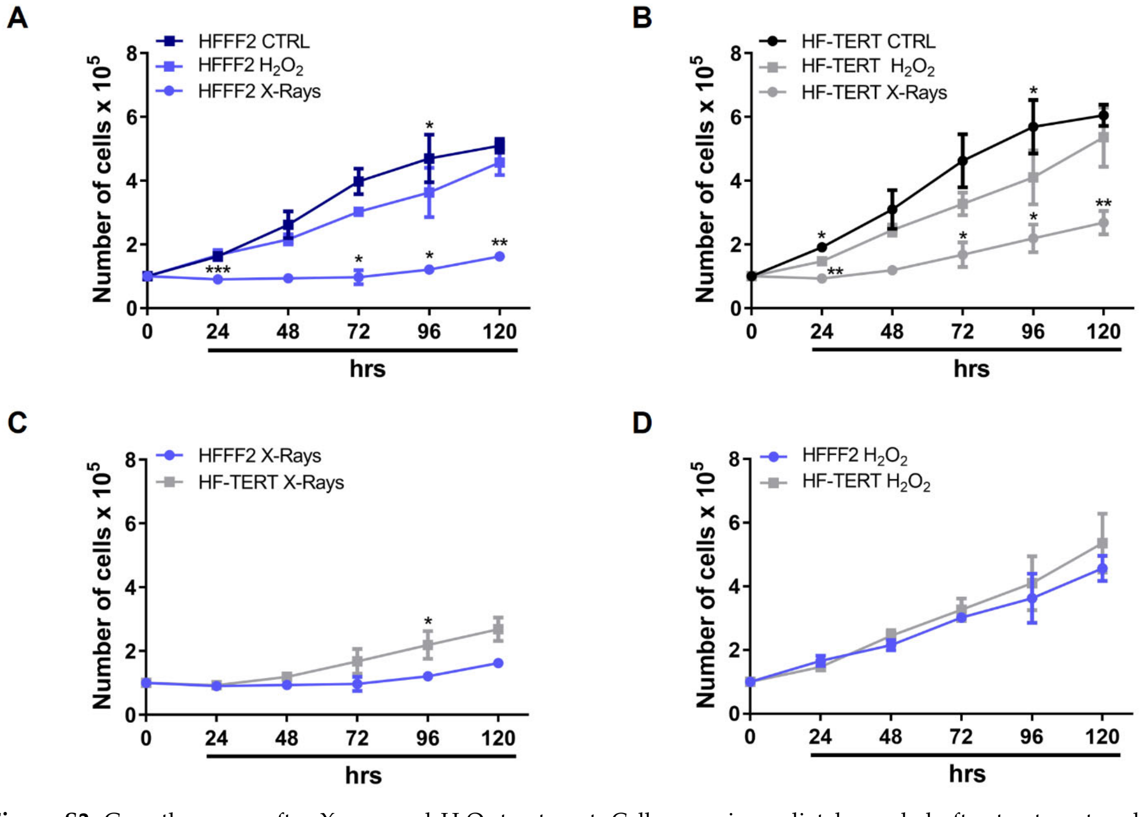

Figure S2. Growth curves after X-rays and H_2O_2 treatment**.** Cells were immediately seeded after treatment and were counted every 24 h until 120 h. (**A**,**B**) The graphs display the growth curves for HFFF2 and HF-TERT cells after 4 Gy of irradiation and 1 hour of hydrogen peroxide treatment. The error bars indicate the mean ± standard error of the mean (SEM). Statistical analysis is performed between treated and untreated cells. * $p < 0.05$; ** $p < 0.01$; *** $p < 0.001$ by t-test. (**C**,**D**) Growth curves of treated cells after X-ray irradiation and H_2O_2 treatment. The error bars indicate the mean ± SEM. Statistical analysis is performed between normal and transduced cells. * $p < 0.05$; by *t*-test.

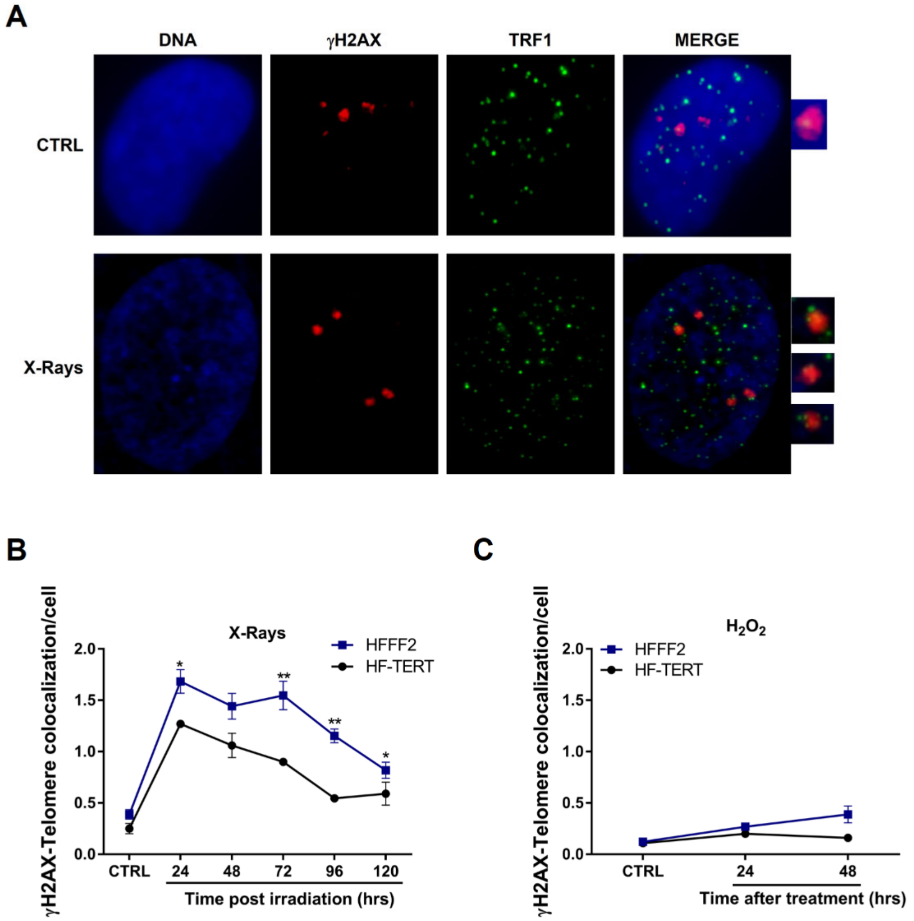

Figure S3. Immunofluorescence of telomere dysfunction-induced foci (TIF) between γH2AX foci (red spot) and telomeric protein TRF1 (green spot). (**A**) The enlarged images show additional foci at telomeres from this and other nuclei. (**B**,**C**) Comparisons of γ-H2AX/TRF1 colocalization in both cell lines after both treatments. Bars represent the mean percentage ± SEM. Statistical analysis is performed between HFFF2 and H-TERT treated samples. * $p < 0.05$; ** $p < 0.01$; by t-test.

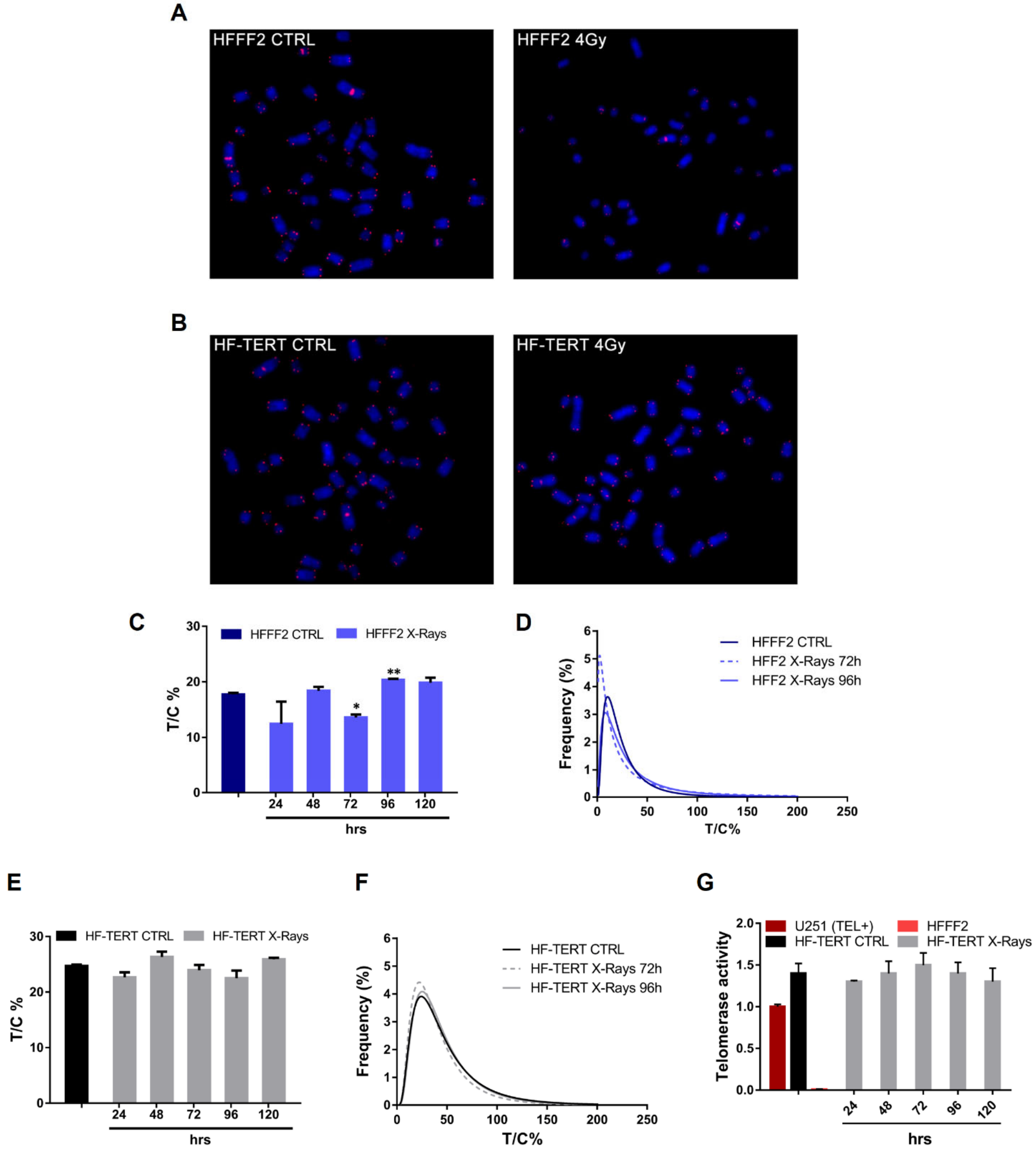

Figure S4. Analysis of telomere length in all samples after X-rays. (**A**,**B**) Example of metaphases of untreated and treated HFFF2 cells (**A**) and untreated and treated and HF-TERT cells (**B**). (**C**) Results of telomere length after X-rays treatment in HFFF2 cells. (**D**) Log-Gaussian distributions of control and irradiated HFFF2 samples at 72 and 96 h since treatment. (**E**) The histogram represents the telomere length after X-irradiation in HF-TERT cells. (**F**) Log-Gaussian distributions of control and irradiated samples of HF-TERT at 72 and 96 h since X-rays. (**G**) Telomerase activity in control and X-rays treated HF-TERT cells. Telomerase-positive cell line (U251MG) is used as positive control and HFFF2 cells are used as negative control. Values are expressed as mean values ± SEM. Statistical analysis is performed between treated and control samples. * $p < 0.05$; ** $p < 0.01$; by *t* Student test.

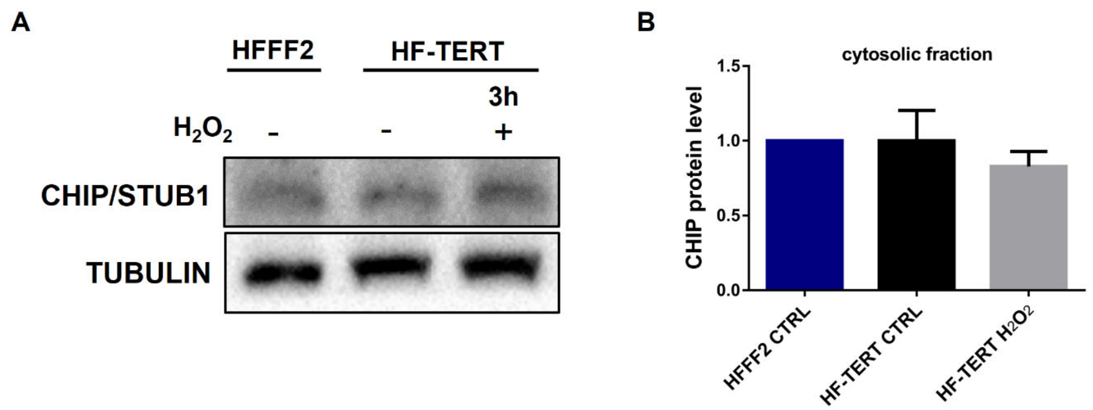

Figure S5. Western blot of CHIP. (**A**) Western blot shows specific bands for cytosol level of CHIP and tubulin, used as control protein in untreated HFFF2 and in untreated and treated HF-TERT cells. (**B**) Cytosolic level of CHIP protein after 3 h since treatment. Data represent mean ± SEM.

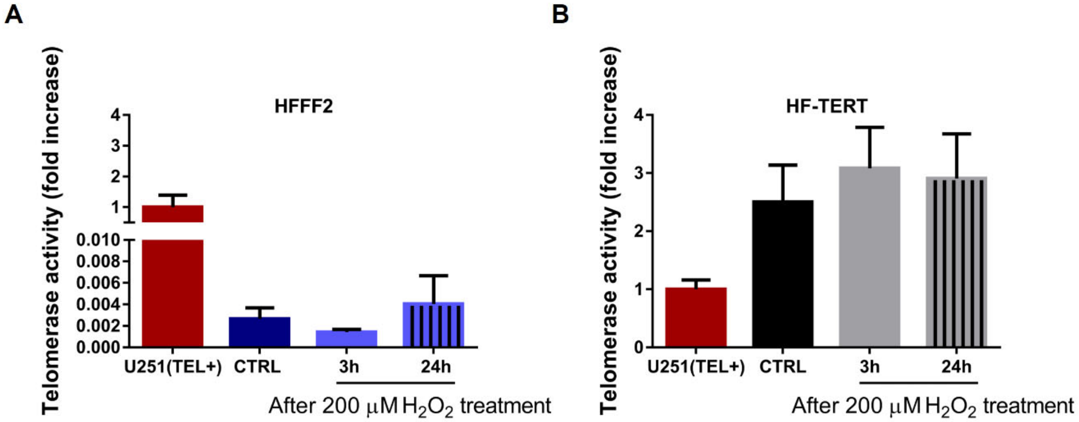

Figure S6. RQ-TRAP assay. (**A**) The data on telomerase activity in untreated and treated fibroblasts after 3 and 24 h post treatment. (**B**) Telomerase activity in untreated and treated hTERT overexpressing fibroblasts after 3 and 24 h since H_2O_2 treatment. Data are normalized to the control value represented by U251MG used as positive control. The error bars indicate the mean ± SEM.

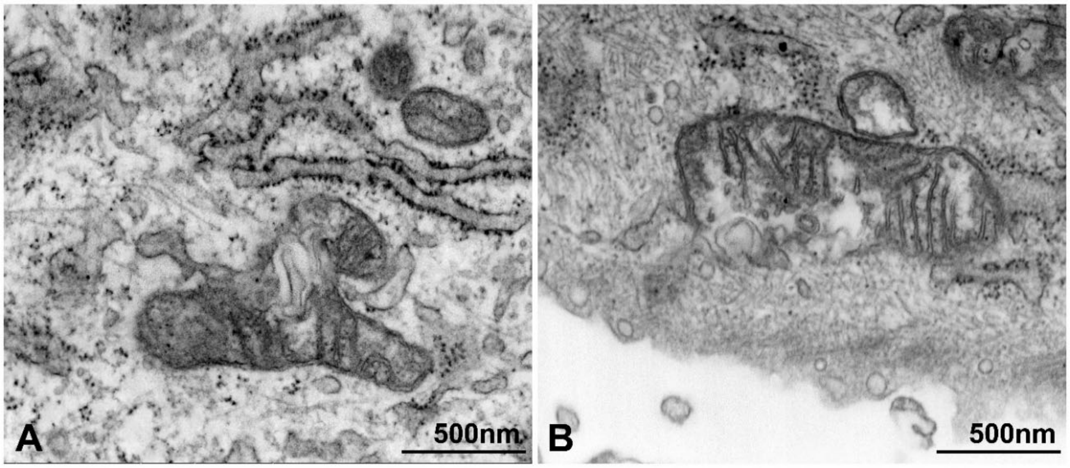

Figure S7. Close up of mitochondria showing outer membrane rupture in HFFF2 cell (**A**) and HF-TERT (**B**) cells following H_2O_2 treatment. Note the severe structural alteration of the organelles, which may assume a branched appearance.

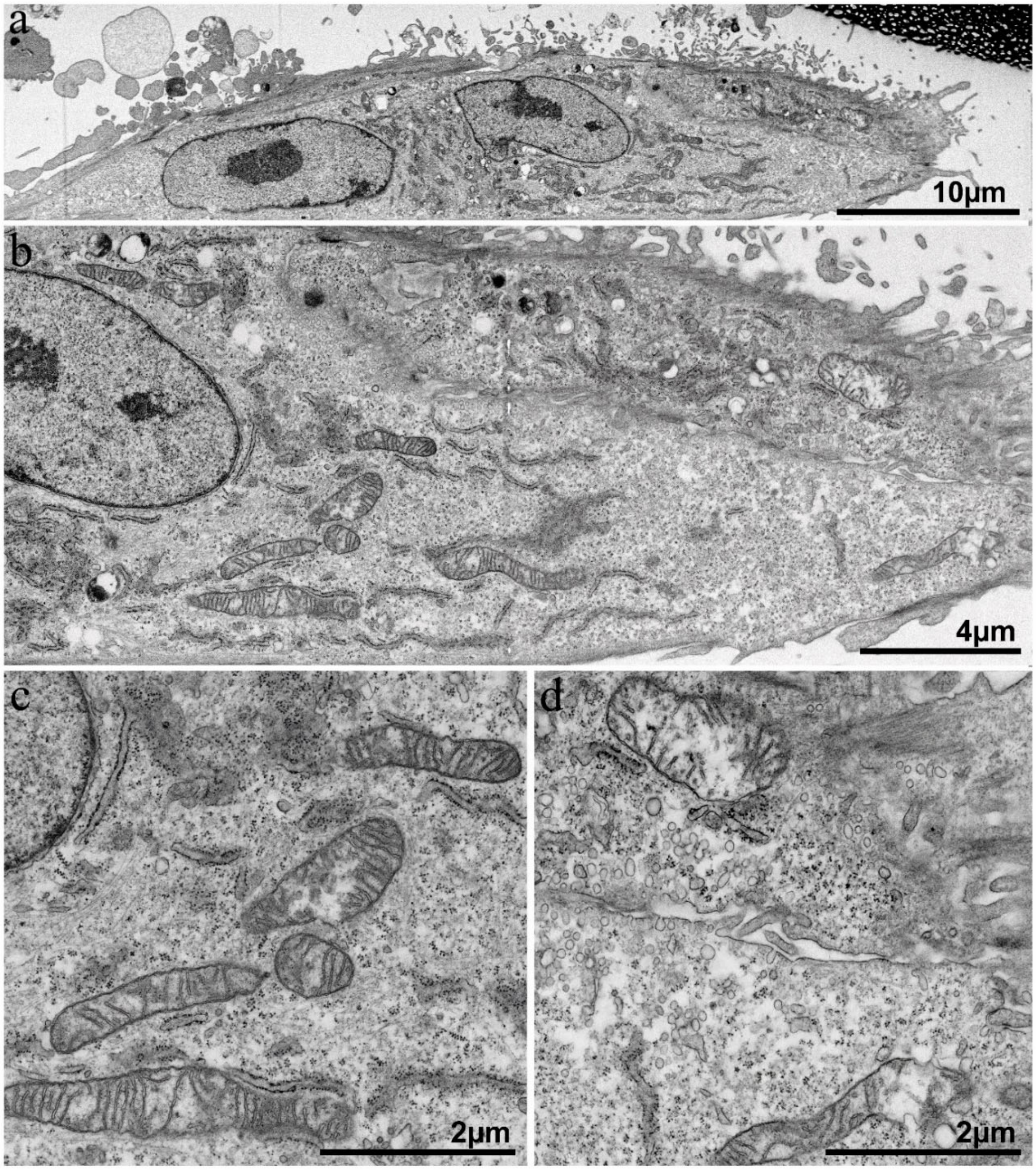

Figure S8. (**A**,**B**) Micrographs with incremental magnification of a HFFF2 cells after hydrogen peroxide treatment. Note in figure (**C**) swelling of the mitochondria and in figure (**D**) the extensive morphological damage in the form of outer membrane rupture and severe loss and fragmentation of the *cristae*.

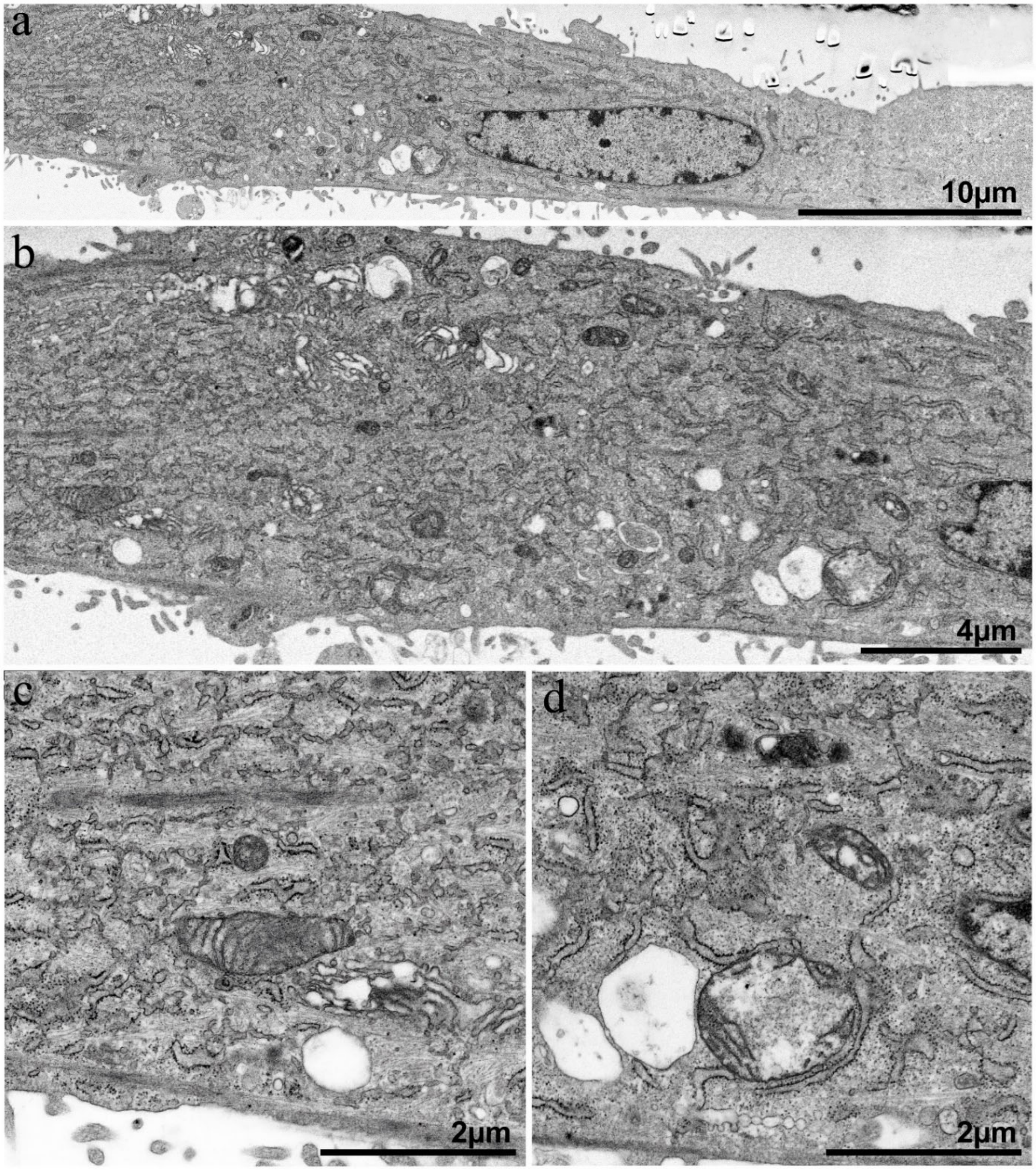

Figure S9. (**A**,**B**) Micrographs with incremental magnification of HF-TERT cells after hydrogen peroxide treatment. Figure (**D**) depicts a strongly swollen mitochondrion, while figure (**C**) shows an organelle with a well-preserved morphology that is similar to those observed in the control cells.
